# How to Diagnose and Risk Stratify Brugada Syndrome. Comment on Matusik et al. Twelve-Lead ECG, Holter Monitoring Parameters, and Genetic Testing in Brugada Syndrome: Insights from Analysis of Multigenerational Family with a History of Sudden Cardiac Arrest during Physical Activity. *J. Clin. Med.* 2023, *12*, 6581

**DOI:** 10.3390/jcm12247548

**Published:** 2023-12-07

**Authors:** Naoya Kataoka, Teruhiko Imamura

**Affiliations:** Second Department of Internal Medicine, University of Toyama, Toyama 930-0194, Japan; nkataoka@med.u-toyama.ac.jp

Brugada syndrome stands as an arrhythmogenic disorder bearing the grim specter of heightened susceptibility to syncopal episodes and sudden cardiac demise. Matusik and associates have undertaken a comprehensive assessment of a multigenerational family originating from a patient whose life was abruptly extinguished via cardiac arrest, ultimately posthumously diagnosed with Brugada syndrome [1]. Their meticulous investigation reveals that the employment of 12-lead Holter electrocardiogram monitoring, complemented by a judiciously tailored placement of precordial leads, is emerging as a valuable diagnostic tool within the domain of Brugada syndrome, fostering personalized risk stratification in the realm of medical practice.

Earlier investigations ventured into multigenerational family analyses pertaining to Brugada syndrome, bearing relevance in the context of screening for suspected Brugada syndrome cases. Nevertheless, it is imperative to discern that their utility may not necessarily extend to the nuanced task of risk stratification concerning fatal arrhythmia in asymptomatic patients [2]. The authors, therefore, warrant scrutiny in evaluating the potential of their findings to enhance risk stratification endeavors.

The authors’ focal point resides in the exploration of the SCN5A mutation, an endeavor that uncovers its presence in a mere fraction, constituting a paltry 20% of patients grappling with Brugada syndrome [1]. Recent scholarship has elucidated the enigmatic link between Brugada syndrome and late gadolinium enhancement localized at the right ventricular outflow tract, as evidenced through cardiac magnetic resonance imaging. This anomaly signifies the potential association of structural maladies with Brugada syndrome, hence impelling the reevaluation of this syndrome as a multifaceted ailment replete with a multitude of etiological factors [3]. The current literature defines this complex ensemble of disorders as sub-epicardial cardiomyopathy, a syndrome that instigates spontaneous ventricular fibrillation [4]. In light of these considerations, family analyses should not be unduly fixated on the gene mutation aspect.

The authors, in their exhaustive exploration, have unearthed several parameters that bear the semblance of a connection to the diagnosis of Brugada syndrome [1]. These encompass P-wave duration, PR interval, and the notorious aVR sign, all intricately entwined with conduction irregularities. Their findings bear a logical plausibility, for conduction disorders are intrinsically linked to the manifestation of Brugada-like electrocardiogram patterns. Nevertheless, it is imperative to recognize that these parameters may not be equally conducive for the task of risk stratification. Recent investigations have underscored the critical role of phase 2 re-entry in precipitating ventricular fibrillation episodes among patients afflicted with Brugada syndrome [3]. This realization obliges us to delineate the distinction between risk stratification and the diagnostic endeavor associated with Brugada syndrome.
